# The Activated AMPK/mTORC2 Signaling Pathway Associated with Oxidative Stress in Seminal Plasma Contributes to Idiopathic Asthenozoospermia

**DOI:** 10.1155/2022/4240490

**Published:** 2022-06-08

**Authors:** Nannan Cao, Chunhui Hu, Bintong Xia, Yan He, Jiaolong Huang, Zhicheng Yuan, Jie Deng, Peng Duan

**Affiliations:** ^1^Postgraduate Union Training Base of Jinzhou Medical University, Xiangyang No. 1 People's Hospital, Hubei University of Medicine, Xiangyang, 441000 Hubei Province, China; ^2^Key Laboratory of Zebrafish Modeling and Drug Screening for Human Diseases of Xiangyang City, Department of Obstetrics and Gynaecology, Xiangyang No. 1 People's Hospital, Hubei University of Medicine, Xiangyang, 441000 Hubei Province, China; ^3^Department of Clinical Laboratory, Xiangyang No. 1 People's Hospital, Hubei University of Medicine, Xiangyang, 441000 Hubei Province, China; ^4^Department of Urology, Xiangyang No. 1 People's Hospital, Hubei University of Medicine, Xiangyang, 441000 Hubei Province, China; ^5^The Fourth Clinical Faculty, Hubei University of Medicine, Shiyan, 44200 Hubei Province, China; ^6^Hubei Key Laboratory of Embryonic Stem Cell Research, Hubei University of Medicine, Shiyan, 442000 Hubei Province, China

## Abstract

Asthenozoospermia is a common form of abnormal sperm quality in idiopathic male infertility. While most sperm-mediated causes have been investigated in detail, the significance of seminal plasma has been neglected. Herein, we aimed to investigate the possible pathogenic factors leading to decreased sperm motility based on seminal plasma. Semen was collected from normo- (NOR, *n* = 70), idiopathic oligo- (OLI, *n* = 57), and idiopathic asthenozoospermic (AST, *n* = 53) patients. Using attenuated total reflection-Fourier transform infrared coupled with chemometrics, distinct differences in the biochemical compositions of nucleic acids, protein structure (amides I, II, and III), lipids, and carbohydrates in seminal plasma of AST were observed when compared to NOR and OLI. Compared with NOR and OLI, the levels of peptide aggregation, protein phosphorylation, unsaturated fatty acid, and lipid to protein ratio were significantly increased in AST; however, the level of lipid saturation was significantly decreased in seminal plasma of AST. Compared with NOR, the levels of ROS, MDA, 8-iso-prostaglandin F2*α* (8-isoPGF2*α*), and the ratio of phospho-AMPK*α*/AMPK*α*1 were significantly increased in AST; however, the levels of SOD, glutathione S-transferase (GSTs), protein carbonyl derivative (PC), and the ratio of phospho-Rictor/Rictor were significantly decreased in seminal plasma of AST. Changes of the AMPK/mTORC2 signaling in the seminal microenvironment possibly induce abnormal glucose and lipid metabolism, which impairs energy production. Oxidative stress potentially damages seminal plasma lipids and proteins, which in turn leads to impaired sperm structure and function. These findings provide evidence that the changes in seminal plasma compositions, oxidative stress, and activation of the AMPK/mTORC2 signaling contribute to the development of asthenozoospermia.

## 1. Introduction

Asthenozoospermia was defined as progressive motility < 32% or total motility < 40% after two to seven days of sexual abstinence. The common causes include age, lifestyle changes, inflammation, exposure to chemical pesticides, electromagnetic radiation, and air pollution [1–3]. These factors can lead to abnormal semen coagulation and liquefaction, immune infertility, endocrine disorders, abnormal sperm structure, abnormal chromosomes, and varicocele, thus leading to a decrease in sperm motility [4, 5]. However, in addition to these causes, 30–40% of asthenozoospermia cases still remain unexplained and are called idiopathic asthenozoospermia (AST).

As an important component of semen, seminal plasma contains various biochemical molecules and nutrients, and it provides spermatozoa with a stable and safe microenvironment to perform physiological functions [6–8]. Notably, proteins extracted from seminal exosomes could prompt sperm capacitation through increased induction of tyrosine phosphorylation, therefore inducing the acrosome reaction [9, 10]. For example, when compared to normozoospermic men, the levels of some exosomal proteins, such as SPAG11B and CRISP1, were changed in the seminal plasma of asthenozoospermic men [9, 10]. The seminal exosomal SPAG11B was found to be involved in sperm maturation [11]. The lack of seminal exosomal CRISP1 was associated with impaired sperm capacitation and an acrosome reaction [9]. Seminal oxidative stress and abnormal energy metabolism are the main causes affecting sperm motility [6, 12]. The AMPK/mTOR pathways have been shown to regulate energy homeostasis and be activated in response to oxidative stress [13–18]. An increasing number of studies have implicated that AMPK and mTOR signaling in the seminal microenvironment plays a contributing role in spermatogenesis, sperm maturation, capacitation, and the acrosome reaction [13, 14, 17, 19–22]. Alternations of seminal components relate to sperm motility, but the specific mechanism of its effect on the development of asthenozoospermia still needs to be further explored.

With the advantages of nondestructive, relatively reagent-free methods that can generate rapid, high-throughput, and robust results in real time, biospectroscopy techniques exhibit high sensitivity to minimal changes within biomolecules [23]. As a normal biospectroscopy technique, attenuated total reflection-Fourier transform infrared (ATR-FTIR) spectroscopy is fast and easy-to-use, with minimal sample preparation, is a simultaneous analysis of a wide range of different biomolecules, and preserves the integrity of samples [24–26]. ATR-FTIR has also been widely applied to distinguish normal and pathological conditions by detecting cells, tissues, or biofluids, such as serum and seminal plasma [26–28]. In addition, ATR-FTIR spectroscopy is capable of analyzing biomolecules, such as lipids, proteins, carbohydrates, and nucleic acids, thus providing metabolic profiles that help to explain disease pathogenesis [26, 29, 30]. In this study, ATR-FTIR spectroscopy coupled with chemometric analysis was successfully employed to detect biochemical variations of interest between AST and NOR/OLI. Moreover, seminal biomarkers associated with oxidative stress and target proteins in the AMPK/mTOR pathway were analyzed to provide some insights into the molecular events leading to AST. The aim of this study was to explore the pathogenesis of asthenozoospermia by detecting biochemical alterations in seminal plasma regulating sperm motility.

## 2. Materials and Methods

### 2.1. Subjects

Male patients undergoing assisted reproductive techniques were recruited in this study and closely followed for at least four weeks. Each patient received a unified medical history inquiry. Sociodemographic data and information on smoking habits, drinking habits, sedentary time, sleep duration, and body mass index (BMI) were collected via a self-reporting questionnaire. Prior to the study, all included patients understood the purpose of this study and provided written informed consent for data collection. This work was reviewed and acquired the approval of the Ethics Committee of Hubei University of Medicine (approval no. 2019-TH-013).

### 2.2. Inclusion and Exclusion Criteria

A total of 180 patients, aged 20-40 years, without any other systemic diseases were enrolled in the study. Participants were evaluated for semen parameters and divided into three groups: 70 men with normozoospermia (NOR), recruited as normal controls; 57 men with idiopathic oligozoospermia (OLI), recruited as positive controls; and 53 men with idiopathic asthenozoospermia (AST), recruited as the case group. Exclusion criteria were as follows: varicocele, cryptorchidism, chromosomal disorders, congenital malformations, or infections of the reproductive system. Participants who were taking drugs that impair spermatogenesis and sperm motility (antitumour, antiepileptic, antirheumatism, cortin, etc.) and those with malignancy, mental or psychological abnormalities, and habits of serious smoking, heavy drinking, or drug abuse were excluded. Additionally, those with a risky occupation, such as those professions with exposure to radiation, electric welding, high temperatures, or highly polluted operations, were not enrolled.

### 2.3. Semen and Reproductive Hormone Analysis

Fresh semen samples were collected within the sterile plastic jars from every participant by masturbation after 3-7 days of sexual abstinence. The volume of the sample was 3.5 mL ± 1.5 mL. The length of abstinence prior to sampling was documented. Fresh semen samples collected within 1 h after ejaculation were used for routine semen analysis and seminal plasma preparation, and some were kept frozen at -80°C prior to further DNA fragmentation index analysis. Semen analysis was performed according to the *World Health Organization (WHO) Laboratory Manual for the Examination and Processing of Human Semen* (5th edition, 2010) [31]. After collection, the sample was liquefied at 37°C for 20 min before routine analysis and maintained at 37°C during the assessment.

The collected semen volume was measured by using a calibrated test tube. Afterwards, the sperm concentration was checked by using a Bürker chamber and adjusted to 15-20 × 10^6^ sperm/mL using the semen extender. Sperm motility was evaluated by using a computer-aided semen analysis (CASA) system (model S-3, BEIONMED, Shanghai, China). 5 *μ*L of well-mixed, homogeneous sample was applied to each chamber of the Leja slide **(**BEION S-1024, chamber depth 10 *μ*m, BEIONMED, Shanghai, China). The Leja slide was placed on a warm CASA stage at 37°C for 30 s and then analyzed within 5 min. A total of nine sperm kinetic parameters were obtained by analyzing six random fields: curvilinear velocity (VCL), mean angle of deviation (MAD), linearity (LIN), straight line velocity (VSL), amplitude of lateral head displacement (ALH), wobble (WOB), average path velocity (VAP), beat cross frequency (BCF), and straightness (STR). The setting parameters were as follows: frames per second (30) and minimum frames acquired (24). At least 200 sperm cells were counted for motility assessment. Sperm morphology was visualized by using a Diff-Quick staining kit (Bred-015, BRED Life Science Technology Inc., Shenzhen, China) according to the manufacturer's instructions. A minimum of 100 sperm were counted for each assay. Serum hormone concentrations of follicle-stimulating hormone (FSH), luteinizing hormone (LH), and testosterone (T) were analyzed by the automated chemiluminescence system using Beckman Coulter kits (FSH, 33520; LH, 33510; and T, 33560) on the Beckman Coulter brand DxI 800 autoanalyzer.

### 2.4. Determination of Sperm DNA Fragmentation Index and Acrosin Activity

Sperm DNA fragmentation index (DFI) was assessed by the sperm chromatin structure assay (SCSA). The SCSA kit (CP0101-10T) was purchased from Zhejiang CellPro Biotech Co., Ltd. (Ningbo, China). The test was operated strictly according to the manufacturer's instructions. Briefly, the frozen semen samples were placed in a water bath at 37°C until liquefied. An appropriate amount of semen was diluted to a concentration to 2 × 10^6^/mL by TNE buffer (0.01 M Tris-HCL, 0.15 M NaCl, and 1 mM EDTA) to adjust the semen density. 100 *μ*L of the dilution was added to a tube and mixed with 100 *μ*L of acid solution (0.1% Triton X-100, 0.15 M NaCl, and 0.08 M HCL, pH 1.2) for 30 s. The sample was then stained with 300 *μ*L of acridine orange (AO) staining solution (CellPro Biotech Co., Ltd., Ningbo, China) and incubated for 3 min. Then, the sperm DFI was calculated by assessing the ratio of red to total fluorescent cells using a flow cytometer (model EPICSXL, Beckman Coulter, Fullerton, CA, USA). Approximately 10,000 spermatozoa were acquired for each sample at a flow rate of 200-300 events/s.

Sperm acrosin activity was determined by calcium ionophore A23187 with a commercially available kit (Bred-001, BRED Life Science Technology Inc., Shenzhen, China). Briefly, 1 mL liquefied semen was mixed thoroughly with 4 mL 0.9% NaCl and then centrifuged at 2000 r for 5 min. 100 *μ*L aliquot sperm suspension samples after swim-up was incubated for 3 h at 37°C at 5% CO_2_ to induce capacitation. After being recentrifuged and resuspended, the capacitated spermatozoa were then mixed with 2.5 *μ*L of A23187 stock solution in the tube at a final concentration of 10 *μ*mol/L and incubated at 37°C for 1 h. The mixture was then centrifuged at 2000 r for 5 min, and 10 *μ*L of the aliquots was removed to a slide and then the spermatozoa were fixed with 50 *μ*L of 3% (*v*/*v*) glutaraldehyde. Fixed spermatozoa were smeared in duplicate and stained with PSA-FITC for 60 min at 37°C. After rinsing the samples and air-drying, we counted at least 400 spermatozoa in each smear and categorized acrosomes. Acrosome activity was calculated as AR%.

### 2.5. Preparation and Determination of Seminal Plasma

After semen analysis, seminal plasma from the rest of the sample was centrifuged at 3,500 r/min at 4°C for 30 minutes. Subsequently, the supernatant was centrifuged at 14,000 r/min at 4°C for 30 minutes to remove cell debris, and the resulting semen supernatant was aliquoted into new tubes (50 *μ*L per tube) and kept frozen at -80°C prior to analysis.

Seminal neutral *α*-glucosidase (NAG) activity was determined by a modification of Cooper using a commercial kit (Bred-007, BRED Life Science Technology Inc., Shenzhen, China). Briefly, p-nitrophenyl glucopyranoside (PNPG) was converted to p-nitrophenol (PNP) by the enzyme, and PNP was converted to yellow substrate; the absorbance of which was read at 405 nm by using an automated ELISA analyzer (model 2900, BRED Life Science Technology Inc., Shenzhen, China), after being added with 0.1 M Na_2_CO_3_. The concentration of PNP was determined by using the standard curve which represented NAG activity. The results of NAG activity were expressed as mU/ejaculate.

Elastase levels in the seminal plasma were tested by commercial enzyme-linked immunosorbent assays (Bred-006, BRED Life Science Technology Inc., Shenzhen, China). The elastases in seminal plasma, after binding with the microplate-coated elastase monoclonal antibodies, react with the specific antibody marked by the horse radish peroxidase (HRP) and finally form the elastase-*α*1-PI complex. The absorbance of the chromogenic product was read photometrically at 450 nm by using an ELISA analyzer. The concentration of seminal plasma elastase shows a positive correlation with the substrate staining strength. Testing was performed strictly according to the manufacturer's instructions.

Seminal plasma zinc concentration was assessed by using a commercial kit (Bred-004, BRED Life Science Technology Inc., Shenzhen, China). The frozen seminal plasma samples were thawed and mixed well on a vortex mixer. After being deionised, 50 *μ*L of the deionised seminal plasma and standards was added to a microplate; then, 200 *μ*L of colour reagent was added and mixed thoroughly. Then, the mixture was placed at room temperature for 1 min. The absorbance of the sample solutions was read at 560 nm by using an ELISA analyzer. The calculated formula of sample zinc concentrations was as follows: seminal plasma zinc (*μ*mol) = zinc concentration (mmol/L) × semen volume (mL).

Seminal plasma fructose was measured using a commercially available Fructose Assay Kit (Bred-010, BRED Life Science Technology Inc., Shenzhen, China) according to the manufacturer's instructions. Briefly, 50 *μ*L of seminal plasma was mixed thoroughly with the deproteinized solutions about 250 *μ*L of ZnSO4 and 100 *μ*L of NaOH. After 10 min of incubation at room temperature, it was centrifuged at 3000g for 10 min. And 200 *μ*L of clear supernatant was mixed with the reagent composed of hexokinase, fructose-6-phosphate dehydrogenase, NAD+, and ATP. The mixture was incubated at 37°C for 15 min. The absorbance of the sample solutions was read at 340 nm by using an ELISA analyzer. The calculated formula is fructose volume per ejaculation (*μ*mol) = fructose concentrations in seminal plasma (mmol/L) × total volume of the semen per ejaculation (mL).

### 2.6. ATR-FTIR and Multivariate Analysis

Prior to spectral analysis, the frozen semen supernatant was thawed in the fridge at 4°C for an average time of 1 hour. After frozen samples were thawed, 30 *μ*L of semen supernatant from one individual patient was deposited on an IR-reflective glass slide (MirrIR Low-E slides; Kevley Technologies). Prepared slides were left to air-dry for a minimum of 2 h prior to spectroscopic examination. Raw infrared spectra were acquired using a Bruker Vector 70 FTIR spectrometer equipped with a Helios ATR attachment containing a single reflection diamond crystal (Bruker Optics Ltd., Coventry, UK). Infrared spectra were recorded with the ATR mode in the spectral range of 4000–800 cm^−1^ at an 8 cm^−1^ resolution with 64 scans coadded. To adjust for heterogeneity within each sample, more than 10 IR spectra were acquired from each of the 10 independent locations across each sample [26]. To compensate for atmospheric and synchrotron ring current changes, a background spectrum was taken before the analysis of each sample [26, 29]. The ATR diamond crystal was cleaned with distilled water and then dried with soft tissue before the acquisition of the spectral background.

Raw infrared spectra obtained from semen samples were converted to absorbance using Bruker OPUS 7.0 software (Bruker Optics, Germany). Both preprocessing and computational analysis of the spectra data were performed using an in-house-developed IRootLab toolbox (http://trevisanj.github.io/irootlab/) running on MATLAB r2017a (The Mathworks Inc., Natick, MA, USA). All spectra were cut to the spectral ranges of 1800–900 cm^−1^ (biofingerprint region) and 3100–2800 cm^−1^ (the lipid CH stretching region). To account for nonbiological differences (e.g., sample thickness and ATR diamond contact pressure) during measurements, the spectra in the region of 1800–900 cm^−1^ were baseline corrected and normalized to amide I (1650 cm^−1^), and the spectra in the region of 3100–2800 cm^−1^ were baseline corrected and vector normalized [32].

Computational analysis was applied to the preprocessed and mean-centred spectral data. Multivariate classification by means of cross-validated PCA-LDA (leave-one-out cross-validation) was employed to discriminate AST from NOR and OLI [26, 29]. The output data derived from PCA-LDA were extracted and represented in the form of 3-D score plots and cluster vectors [33]. The 3-D score plots were used to describe the similarities and differences between the groups (AST *vs.* NOR/OLI). As scores on linear discriminant 1 (LD1) and linear discriminant 2 (LD2) spaces contained most of the variance in the spectral data, the difference of the distance in LD1 and LD2 space can be used to demonstrate biochemical changes between the groups [29, 32]. Cluster vectors from PCA-LDA were developed to extract the top ten most prominent wavenumber biomarkers that mainly contribute to category segregation and reveal biochemical intergroup differences. These extracted wavenumber biomarkers were subsequently investigated for relative changes in absorbance intensity between the groups. Moreover, these discriminating spectral wavenumbers were tentatively assigned to different biomolecules matched to previously identified spectral biomarkers. For more detailed chemometric analysis applied in ATR-FTIR spectroscopy, interested readers are directed to these references [26, 30, 32, 33].

### 2.7. Determination of Indicators Related to Oxidative Stress in Seminal Plasma

Prior to analysis, the frozen semen supernatant was thawed in the fridge at 4°C for an average time of 1 hour. The levels of superoxide dismutase (SOD) (no. A001-3-2), glutathione S-transferases (GSTs) (no. A004-1-1), and malondialdehyde (MDA) (no. A003-1-2) were tested by using ELISA kits (Nanjing Jiancheng Bioengineering Institute, China). The level of reactive oxygen species (ROS) was determined by the luminol chemiluminescence assay using a commercial kit (GMS 14036.2, Genmed, Shanghai, China). The levels of protein carbonyl derivatives (PC) and 8-iso-prostaglandin F2*α* (8-isoPGF2*α*), which represented the degree of oxidative damage in protein and lipids, respectively, were individually determined by using ELISA kits (STA-310 and STA-337, Cell Biolabs, San Diego, CA, USA). A quality control, measurement curve, and operations were strictly followed with the manufacturer's instructions for the kits. The experiments above were repeated at least three times.

### 2.8. Simple Western Assay (WES)

Prior to analysis, the frozen semen supernatant was thawed in the fridge at 4°C for an average time of 1 hour. The semen supernatant was mixed with ice-cold acetone in a 1 : 4 ratio and incubated overnight at 4°C to precipitate proteins. The subsequent protein precipitate was washed three times with ice-cold acetone. Thereafter, the total protein was extracted from the protein precipitate using ice-cold lysis buffer at a ratio of 1 : 2. The extract was centrifuged at 12,000 × *g* for 5 min at 4°C to remove any precipitate and collect the supernatant. Protein content in the supernatant was detected by using the Bradford Protein Assay Kit (Beyotime, Shanghai, China).

Capillary western blot analysis was performed using the ProteinSimple™ WES automatic system and in accordance with the manufacturer's instructions (ProteinSimple™, Santa Clara, CA, USA). For the detection of phospho-Rictor (Ser1219) and Rictor, samples were run on a 66–440 kDa WES separation module (ProteinSimple SM-W008). For detection of phospho-Raptor (Ser792), Raptor, phospho-AMPK*α* (Thr172), AMPK*α*1, glycogen synthase 1, PFK-1, glucose transporter GLUT1 (Glut1), lactate dehydrogenase (LDH), MCT4, and *β*-actin, samples were run on a 12–230 kDa WES separation module (ProteinSimple SM-W004). The concentration of phosphorylated protein is 2 *μ*g/*μ*L, and the concentration of other proteins is 1 *μ*g/*μ*L. Protein samples (10 *μ*L) were mixed with the Fluorescent Master Mix and heated at 95°C for 5 min. The ladder, blocking reagent (antibody diluent), primary antibodies, streptavidin-HPR, HPR-conjugated secondary antibodies, and chemiluminescent substrate were added to designated wells in an assay plate. The prepared assay plate was placed into the WES instrument, followed by the insertion of capillaries. The bands were quantified detected for three times. The western blot images were generated and quantified using Compass software after normalization by *β*-actin (loading control).

The antibody against phospho-Rictor (Ser1219) (Millipore, cat. no. 07-1331, dilution 12.5 *μ*g/mL), Rictor (CST, cat. no. 2114S, dilution 1 : 100), phospho-Raptor (Ser792) (CST, cat. no. 2083S, dilution 1 : 100), Raptor (Ser792) (CST, cat. no. 2280S, dilution 1 : 100), phospho-AMPK*α* (Thr172) (CST, cat. no. 2535S, dilution 1 : 100), AMPK*α*1 (Abcam, cat. no. ab32047, dilution 1 : 200), Glut1 (Abcam, cat. no. ab115730, dilution 1 : 500), LDH (Abcam, cat. no. ab52488, dilution 1 : 500), glycogen synthase 1 (SANTA, cat. no. sc-81173, dilution 1 : 100), PFK-1 (SANTA, cat. no. sc-67028, dilution 1 : 100), MCT4 (SANTA, cat. no. sc-50329, dilution 1 : 200), and *β*-actin (Santa Cruz, cat. no. sc-4778, dilution 1 : 100).

### 2.9. Statistical Analyses

All analyses were performed using SPSS version 12.0 (SPSS, Chicago, IL, USA) and GraphPad Prism 4.0 (GraphPad Software, La Jolla, California, USA). Continuous variables were summarized as the median (interquartile range [IQR]). Qualitative data were expressed as frequencies and percentages. The box plot diagram was used to visualize the distribution of the datasets. Statistical analyses were performed using either an independent-sample *t*-test, chi-squared test, or one-way analysis of variance (ANOVA), followed by Fisher's least significant difference (LSD) test and Dunnett's T3 post hoc test. A value of *P* < 0.05 was considered statistically significant in all statistical tests.

## 3. Results

### 3.1. Characteristics of the Subjects

For this study, 70 men with NOR, 57 men with OLI, and 53 men with AST were recruited. The semen type and sociodemographic and clinical characteristics of these subjects are summarized in Supplemental Table 1. There was no significant difference in age, BMI, education, smoking habit, drinking habit, sedentary time, or sleep duration among NOR, OLI, and AST (*P* > 0.05).

Semen from the three groups was collected to compare clinical characteristics. Compared with NOR, AST exhibited significantly lower levels of sperm concentration, motility percentage, LIN, VSL, WOB, VAP, BCF, STR, normal morphology, acrosome-reacted sperm, and acrosin (*P* < 0.05). Furthermore, the levels of MAD and DNA fragment index were dramatically increased in AST compared to NOR (*P* < 0.05). However, there was no significant difference in ejaculate volume, VCL, ALH, NAG, elastase, Zn, or fructose between NOR and AST (*P* > 0.05). Moreover, the levels of serum FSH, LH, and T between NOR and AST were not significantly different (*P* > 0.05).

Compared with NOR, OLI patients had a lower ejaculate volume, sperm concentration, total sperm count, total motility percentage, progressive motility percentage, LIN, VSL, WOB, BCF, STR, normal morphology, and acrosome-reacted sperm (*P* < 0.05). Furthermore, the level of DNA fragment index was distinctly increased in OLI compared to NOR (*P* < 0.05). However, there was no significant difference in nonprogressive motility percentage, VCL, MAD, ALH, VAP, NAG, elastase, Zn, fructose, or acrosin between NOR and OLI (*P* > 0.05). Moreover, the levels of serum FSH, LH, and T between NOR and OLI were not significantly different (*P* > 0.05).

In comparison with OLI, AST exhibited a significantly lower motility percentage and acrosin (*P* < 0.05). Furthermore, compared to OLI, sperm concentration, total sperm count, normal morphology, and DNA fragment index were evidently increased in AST (*P* < 0.05). However, there was no significant difference in ejaculate volume, VCL, MAD, LIN, VSL, ALH, WOB, VAP, BCF, STR, acrosome-reacted sperm, NAG, elastase, Zn, or fructose between AST and OLI (*P* > 0.05). Moreover, the levels of serum FSH, LH, and T between AST and OLI were not significantly different (*P* > 0.05).

### 3.2. Multivariate Analysis of ATR-FTIR Spectral Data

**Figure 1:**
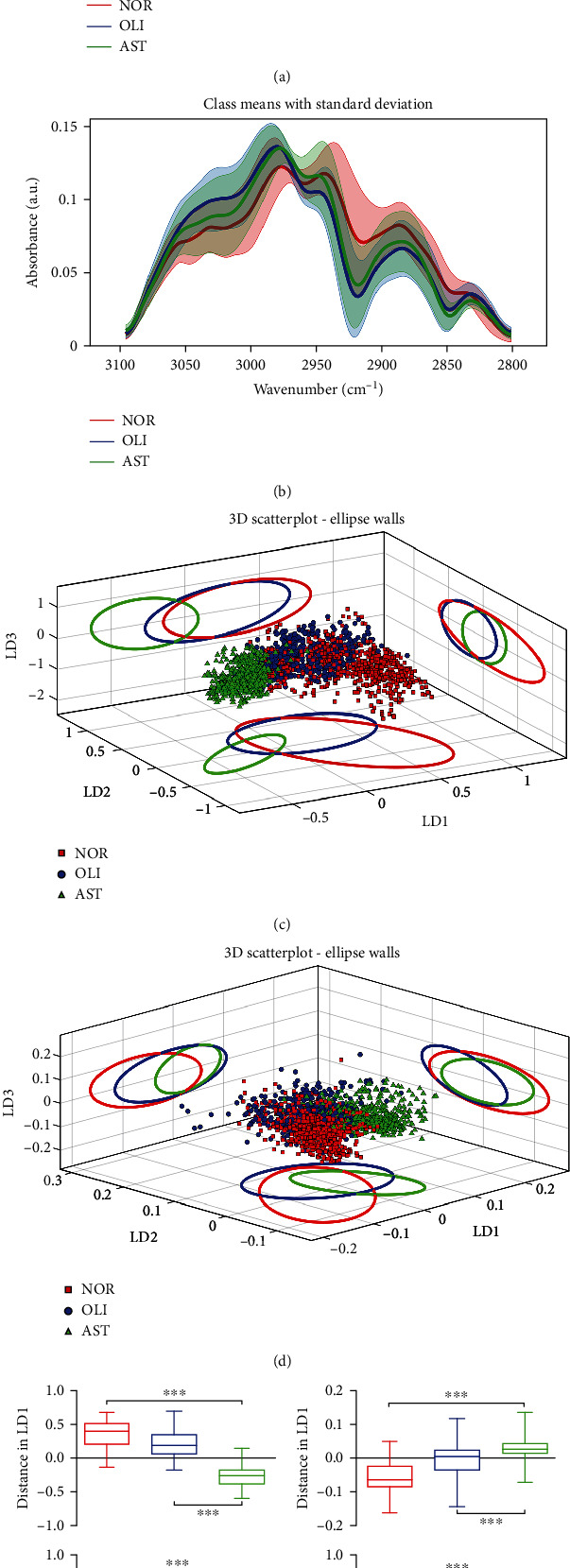
ATR-FTIR spectral classification of AST *vs.* NOR/OLI for seminal plasma samples. Mean preprocessed spectra with the standard deviation for all groups analyzed in the regions of 1800–900 cm^−1^ (a) and 3100–2800 cm^−1^ (b). Three-dimensional (3-D) PCA-LDA scores plots for the spectral regions of 1800–900 cm^−1^ (c) and 3100–2800 cm^−1^ (d). The different categories were classified as NOR (red solid line), OLI (blue solid line), and AST (green solid line). PCA-LDA scores regarding LD1 and LD2 in the regions of 1800–900 cm^−1^ (e) and 3100–2800 cm^−1^ (f). NOR: normozoospermia; OLI: idiopathic oligozoospermia; AST: idiopathic asthenozoospermia. LD1: linear discriminant 1; LD2: linear discriminant 2; LD3: linear discriminant 3. ^∗∗∗^*P* < 0.001*vs.* NOR/OLI.

Following the preprocessing step, multivariate analysis was applied to the ATR-FTIR spectra of biomolecules in seminal plasma of the three groups in the biochemical fingerprint region (1800–900 cm^−1^; Figure 1(a)) and the lipid region (3200–2800 cm^−1^; Figure 1(b)). After PCA-LDA, 3-D score plots were employed as a visual means to classify the differences between groups. Distinct (despite some overlap) segregation between AST *vs.* NOR and AST *vs.* OLI was displayed in both the 1800–900 cm^−1^ (Figure 1(c)) and 3200–2800 cm^−1^ regions (Figure 1(d)).

As a classic algorithm in chemometrics, PCA was used to reduce dimensionality (Supplemental Figure 1). The contribution percentages of PCs 1–5 in the 1800–900 cm^−1^ region were 40.70, 26.90, 16.00, 6.46, and 3.12%, respectively (Supplemental Figure 1A). In the 3100–2800 cm^−1^ region, they were 76.70, 7.72, 4.15, 3.01, and 1.75%, respectively (Supplemental Figure 1B). The dimension-reduced data were linearised and classified by LDA. LD1 and LD2, the top two contribution axes of differences, were used to overall characterize the differences in the spectral data between groups. Whether in the spectral regions of 1800–900 cm^−1^ (Figure 1(e)) or 3200–2800 cm^−1^ (Figure 1(f)), significant differences in LD1 and LD2 were observed between AST *vs.* NOR and AST *vs.* OLI (*P* < 0.001).

**Figure 2:**
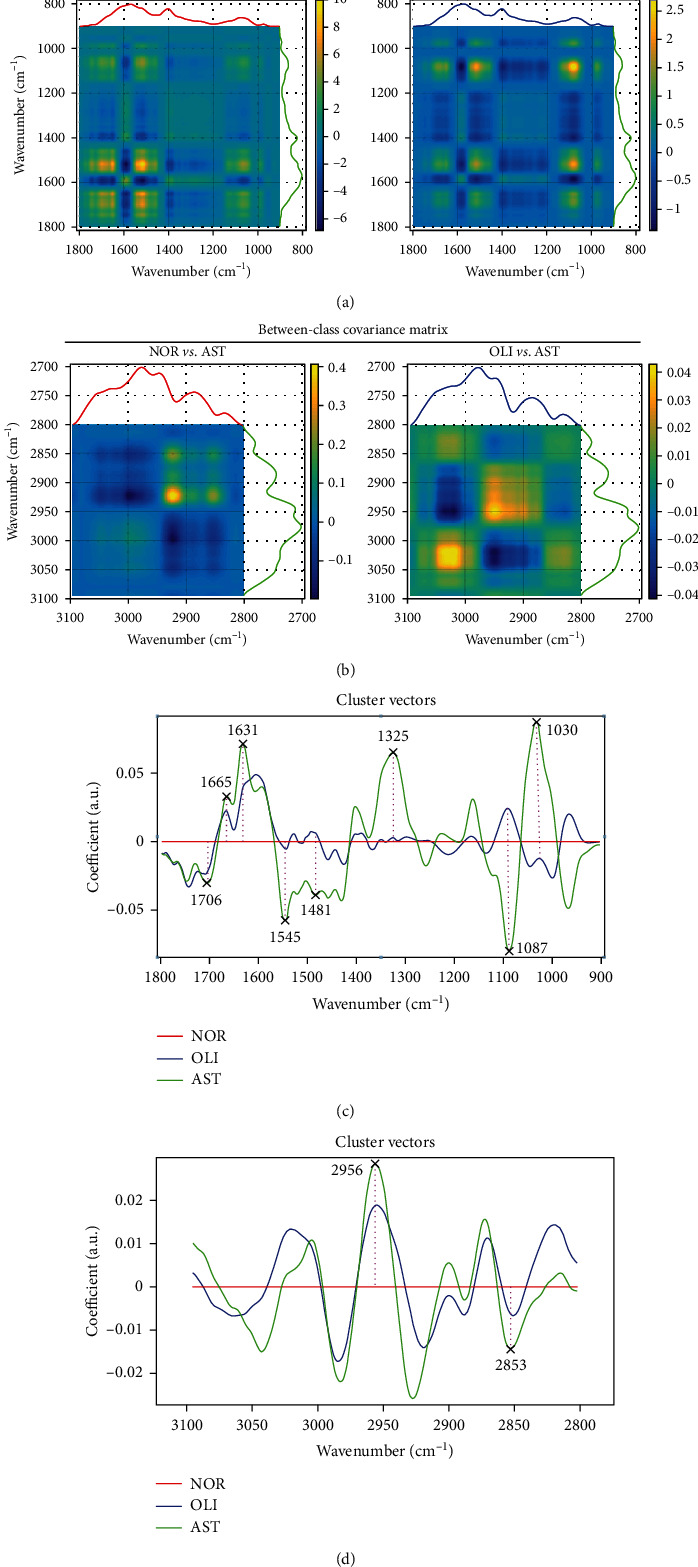
ATR-FTIR spectroscopy coupled with chemometric analysis discriminates AST from NOR/OLI. The between-class covariance matrix map showing the discrimination of AST *vs.* NOR/OLI in the spectral ranges of 3100–2800 cm^−1^ (a) and 1800–900 cm^−1^ (b). Cluster vector plots following PCA-LDA showing the top ten wavenumbers responsible for the discrimination of AST *vs.* NOR/OLI in the spectral ranges 1800–900 cm^−1^ (c) and 3100–2800 cm^−1^ (d). NOR: normozoospermia; OLI: idiopathic oligozoospermia; AST: idiopathic asthenozoospermia.

**Figure 3:**
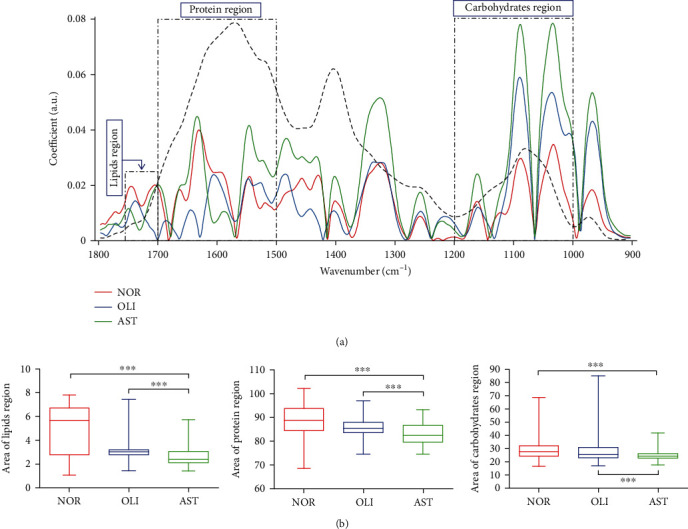
Analysis of the lipid (1750–1700 cm^−1^), protein (1700–1590 cm^−1^), and carbohydrate (1200–1000 cm^−1^) regions. Additive spectral models, showing preprocessed (baseline corrected and normalized to amide I) expected and observed spectra in the biofingerprint region (1800–900 cm^−1^) (a). Comparisons of the lipid, protein, and carbohydrate regions between AST and NOR/OLI (b). NOR: normozoospermia; OLI: idiopathic oligozoospermia; AST: idiopathic asthenozoospermia. ^∗∗∗^*P* < 0.001*vs.* NOR/OLI.

The spectral discrimination of NOR *vs.* AST and OLI *vs.* AST in the spectral ranges of 1800–900 cm^−1^ (Figure 2(a)) and 3100–2800 cm^−1^ (Figure 2(b)) was performed using between-class covariance matrix (BCCM) maps. The prominent yellow and blue spots in BCCM maps represent the spectral region responsible for segregation of AST from NOR and OLI, which were observed in the derived cluster vectors (Figures 2(c) and 2(d)). In the biochemical fingerprint region (Figure 2(c)), the peaks included 1030 (glucose), 1087 (symmetric PO_2_^−^ stretching of DNA and RNA), 1325 (amide *ΙΙΙ*), 1481 (protein conformation), 1545 (amide *ΙΙ*), 1631 (amide *Ι*: *β*-sheet), 1665 (amide *Ι*), and 1706 cm^−1^ (C=O stretching of lipids). In the lipid region (Figure 2(d)), peaks were present at 2853 (*νs.* CH_2_ of lipids) and 2956 cm^−1^ (asymmetric stretching of CH_3_). The peak intensities located at these wavenumbers were significantly different between AST and NOR/OLI (*P* < 0.001), except 1030 cm^−1^ (Supplemental Figure 2). The areas for biomacromolecules were compared among the three groups (Figure 3(a)). Whether compared with NOR or OLI, the lipid (1750–1700 cm^−1^), protein (1700–1590 cm^−1^), and carbohydrate areas (1200–1000 cm^−1^) were significantly decreased in AST (*P* < 0.001; Figure 3(b)).

**Figure 4:**
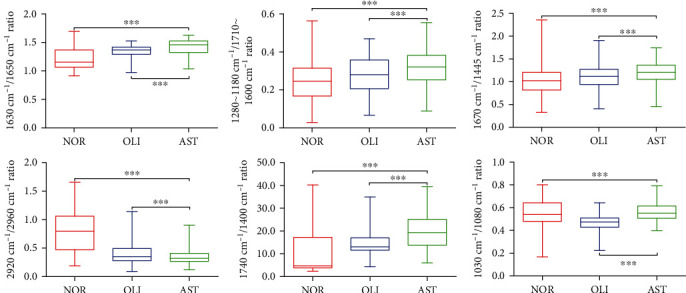
Comparisons of relative intensity ratios of selected ATR-FTIR bands with corresponding tentative biochemical assignments of AST *vs.* NOR/OLI. The 1630/1650 cm^−1^ ratio (peptide aggregation), 1280–1180/1710–1600 cm^−1^ ratio (protein phosphorylation), 1670/1445 cm^−1^ ratio (unsaturated fatty acid level), 2920/2960 cm^−1^ ratio (lipid saturation), 1740/1400 cm^−1^ ratio (lipid to protein), and 1030/1080 cm^−1^ ratio (glycogen to phosphate). NOR: normozoospermia; OLI: idiopathic oligozoospermia; AST: idiopathic asthenozoospermia. ^∗∗∗^*P* < 0.001*vs.* NOR/OLI.

To further explore the alternations of biomacromolecules, we calculated the intensity ratios of selected ATR-FTIR bands corresponding to biochemical assignments. As indicated in Figure 4, compared with NOR, the ratios of 1630/1650 (peptide aggregation), 1280–1180/1710–1600 (protein phosphorylation), 1670/1445 (unsaturated fatty acid), and 1740/1400 cm^−1^ (lipid/protein) were significantly increased in AST (*P* < 0.001). However, the ratios of 2920/2960 (lipid saturation) and 1030/1080 cm^−1^ (glycogen/phosphate) were dramatically decreased in AST (*P* < 0.001). Moreover, in comparison with OLI, AST exhibited significantly higher ratios of 1630/1650, 1280–1180/1710–1600, 1670/1445, 1740/1400, and 1030/1080 cm^−1^ (*P* < 0.001), while the 2920/2960 cm^−1^ ratio was evidently lower in AST (*P* < 0.001).

### 3.3. Oxidative Stress of Seminal Plasma

**Figure 5:**
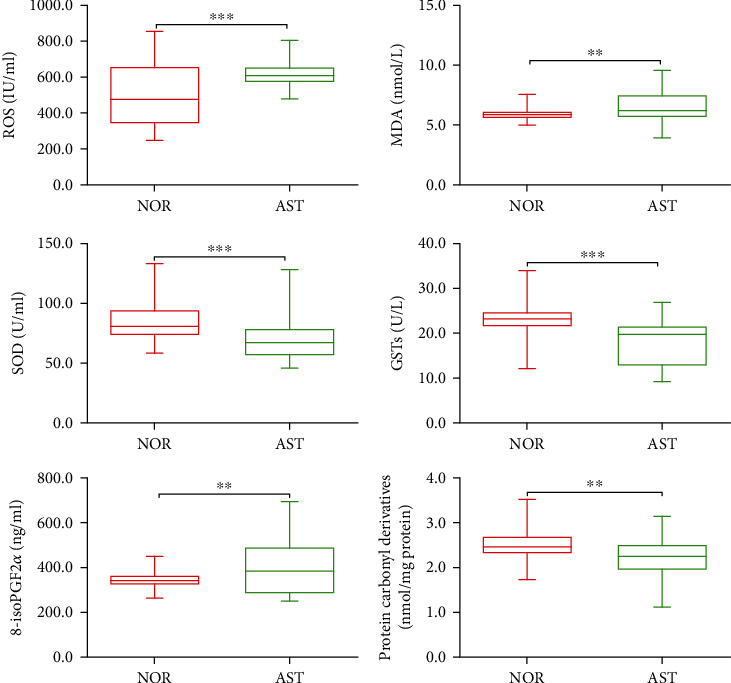
Comparisons of the concentrations of markers related to oxidative stress in the seminal plasma of NOR *vs.* AST. ROS: reactive oxygen species; MDA: malondialdehyde; SOD: superoxide dismutase; GSTs: glutathione S-transferase; 8-isoPGF2*α*: 8-iso-prostaglandin F2*α*; PC: protein carbonyl derivative; NOR: normozoospermia; AST: idiopathic asthenozoospermia. ^∗∗^*P* < 0.01, ^∗∗∗^*P* < 0.001*vs.* NOR.

It has been reported that asthenozoospermia is associated with oxidative stress [34, 35], and the above spectral data showed that the structure and content of lipids and proteins changed; thus, the biomarkers related to oxidative stress of seminal plasma and oxidative damage of biomacromolecules were determined. As indicated in Figure 5, compared with NOR, the ROS and MDA content were significantly increased in AST (*P* < 0.01), while the concentrations of SOD and GST were evidently reduced in AST (*P* < 0.001). Moreover, the level of 8-isoPGF2*α*, a marker of lipid oxidative damage, was evidently increased in AST compared to NOR (*P* < 0.01). The concentration of PC, a marker of protein oxidative damage, was evidently reduced in AST compared to NOR (*P* < 0.01).

### 3.4. Determination of Target Proteins in the AMPK/mTOR Pathway

**Figure 6:**
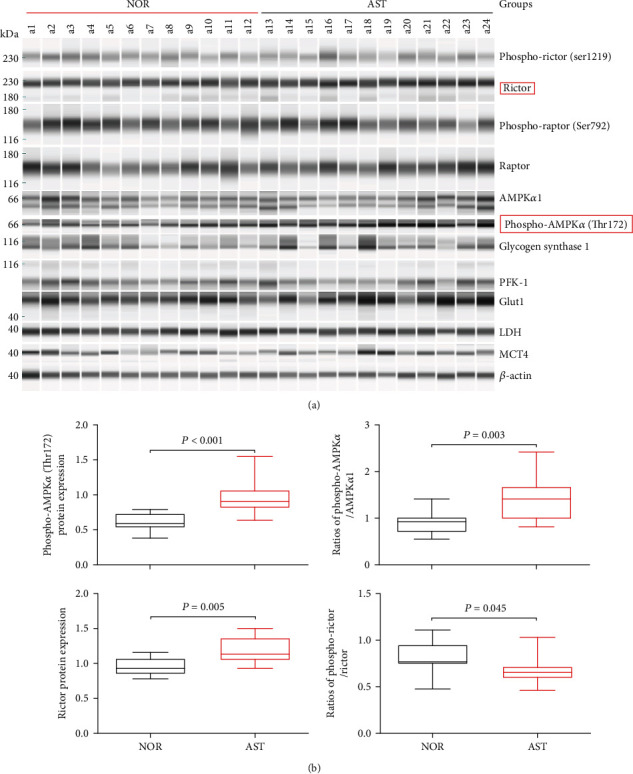
Comparisons of the target proteins expressed in the AMPK/mTOR pathway of NOR *vs.* AST. (a) Protein levels were determined by the ProteinSimple capillary immunoassay. (b) Box plot diagram showing the expression levels of Rictor, phospho-Rictor/Rictor, phospho-AMPK*α* (Thr172), and phospho-AMPK*α*/AMPK*α*1. GLUT1: glucose transporter 1; LDH: lactate dehydrogenase; PFK-1: phosphofructokinase-1; MCT4: monocarboxylate transporter 4; NOR: normozoospermia; AST: idiopathic asthenozoospermia.

AMPK, together with the mTOR signaling pathway, constitutes a switch of anabolism and catabolism. To further explore the relationship between energy metabolism and asthenozoospermia, we determined the target proteins regulating glycolipid metabolism in the AMPK/mTOR pathway. Twelve semen samples with the levels of PC and 8-isoPGF2*α* within the range of P50–P75 were selected from NOR and AST, respectively. The grayscale ProteinSample data from the target proteins in the AMPK/mTOR pathway is displayed in Figure 6(a). As shown in Figure 6(b), in comparison with NOR, the levels of Rictor, phospho-AMPK*α* (Thr172), and the ratio of phospho-AMPK*α*/AMPK*α*1 were significantly increased in AST (*P* < 0.01). Moreover, the ratio of phospho-Rictor/Rictor in AST was evidently decreased (*P* < 0.05). The other target proteins, including phospho-Rictor, Raptor, phospho-Raptor (Ser792), phospho-Raptor/Raptor, AMPK*α*1, glycogen synthase 1, PFK-1, GLUT1, LDH, and MCT4, were not significantly different between NOR and AST (*P* > 0.05; Supplemental Figure 3).

## 4. Discussion

In recent years, with the decline in semen quality caused by various factors, the incidence of male infertility has been progressively increasing. AST is a form without clear causes of human male infertility and is defined by absent or decreasing forward sperm motility. Gene abnormalities and endocrine imbalance are common causes of asthenozoospermia [36–38]. However, the pathogenesis of asthenozoospermia is complex and not yet fully understood. Changes in seminal plasma, an important part of semen, can influence the metabolism, motility, and survival of spermatozoa [6, 7, 34, 35]. Thus, this study investigated the pathogenesis of AST using seminal plasma from patients undergoing assisted reproductive techniques.

Metabolic disturbance in seminal plasma is a common factor leading to lower sperm motility [6–8, 39]. ATR-FTIR is fast, easy-to-use, nondestructive, and noninvasive, and it does not need sample preparation nor any reagent, and it preserves the integrity of samples [24, 25]. In this study, ATR-FTIR spectroscopy was used to detect metabolic perturbations and quantify the extent of the metabolic changes in seminal plasma. Derived from the spectra, the biochemical compositions of nucleic acids, protein secondary structure (amides *Ι* and *ΙΙ*), lipids, carbohydrates, and glucose in seminal plasma were obviously different between AST and NOR/OLI. Seminal plasma is derived from secretions of the seminal vesicles, prostate, testis, epididymis, and bulbourethral and periurethral glands [40]. Seminal plasma compositions, including proteins, lipids, and trace elements, are beneficial to the survival of spermatozoa and could help spermatozoa pass through the female reproductive tract successfully [7]. Changes in every source of seminal plasma could possibly alter seminal plasma compositions, which will alter sperm metabolism, thus relating to abnormal spermatogenesis, sperm maturation, and capacitation and finally affecting sperm motility and fertilization [8]. Our findings indicated that the occurrence of asthenozoospermia was possibly caused by the changes in seminal constituents and spermatozoa metabolism.

Protein is the direct executor of life activities. As a basic component of protein, the state of peptides directly affects the fate of cells and ultimately determines the health conditions or diseases [41, 42]. Previous studies have reported that seminal peptides appear to have a role in sperm motility and thereby fertility [42, 43]. Protein conformational changes, including protein/peptide aggregation, are often triggered by protein phosphorylation [44, 45]. Protein phosphorylation plays a pivotal role in regulating biological processes, such as signal transduction and metabolic regulation, by changing protein conformation, activity, and interaction [45, 46]. We observed that the extent of seminal protein aggregation and protein phosphorylation was much higher in AST than in NOR/OLI. We hypothesize that the degree of seminal protein aggregation is negatively correlated with sperm motility. Sperm motility was possibly affected by the excessive increase in the phosphorylation of seminal proteins, which can also affect the secondary structure of proteins. The effect of seminal protein phosphorylation on sperm motility is worthy of further investigation.

Lipids and other biomolecules, such as protein and peptides in the microenvironment, interact with each other and affect cell membrane fluidity, permeability, and signal transduction [47]. Seminal lipids/fatty acids are expected to play a key role in the membrane structure of spermatozoa, sperm capacitation, sperm metabolism, and the acrosome reaction [39, 48, 49]. Herein, we report that seminal lipids and lipid saturation were lower, but the level of unsaturated fatty acid was higher in AST than in NOR/OLI. These data allow us to hypothesize that the changes in seminal lipids are strongly associated with lower sperm motility. The intensity ratios of lipid/protein and glycogen/phosphate have been considered spectral biomarkers to identify metabolic and compositional alterations [29]. Particularly, the glycogen/phosphate ratio was correlated with cellular metabolic turnover [50]. The present study highlighted a noticeable increase in the ratios of lipid/protein and glycogen/phosphate for AST. We speculate that disturbances in metabolism in the seminal microenvironment could be involved in the development of asthenozoospermia. Once the composition and metabolism of lipids and protein in the seminal microenvironment are altered, the biological and chemical functions of spermatozoa will be impaired or even interrupted, affecting sperm motility [51, 52].

Oxidative stress has been shown to be involved in the pathophysiology of lower sperm motility in asthenozoospermic patients [6, 34, 53]. When oxidative stress occurred, the contents of oxidants increased in seminal plasma, such as ROS, LPO, and MDA, which were harmful to sperm motility, thus leading to asthenozoospermia [54]. High concentration of ROS and deficiency of antioxidants, such as SOD, glutathione peroxidase (GPx), and glutathione reductase (GR), could cause lipid peroxidation and the loss of sperm motility [55]. The level of 8-isoPGF2*α* increased when oxidative stress occurs, which was inversely correlated with semen quality, thus leading to decreased sperm motility [56]. Consistent with previous studies [6, 12, 35, 53], as shown in Figure 5, we found that seminal plasma from patients with AST exhibits excessive oxidative stress, characterized by increased ROS, MDA, and 8-isoPGF2*α* (lipid peroxidation) and decreased antioxidant enzyme activities, such as SOD and GSTs. Interestingly, we also found that seminal PC (oxidative damage of proteins) levels were much lower in AST than in NOR. Our results indicated that when the state of oxidative stress in seminal plasma was changed, oxidative damage occurred in seminal proteins and lipids, impairing the structure and function of sperm [12, 57–59]. Therefore, we hypothesize that changes in seminal oxidative stress are one of the main causes leading to asthenozoospermia.

AMPK signaling is known to be activated by oxidative stress and involved in the motility parameters of human spermatozoa [13–16, 60]. AMPK is a heterotrimeric protein, which consists of an alpha (*α*) catalytic subunit, a scaffolding beta (*β*) subunit, and a regulatory gamma (*γ*) subunit. Lower levels of ATP favor the phosphorylation at Thr172 (catalytic *α* subunit) and subsequently lead to AMPK activation [13]. In this study, the presence of AMPK proteins has been confirmed in human seminal plasma. Moreover, a significant increase was found in AMPK phosphorylation and the ratio of phospho-AMPK*α*/AMPK*α*1 in seminal plasma of AST. We hypothesize that AMPK signaling plays an important role in the interplay between spermatozoa functions and their microenvironment, which potentially have negative impacts on human sperm motility [13]. mTOR, which is one of the major downstream effector of AMPK, integrates both intracellular and extracellular signals and mediates energy supply, lipid metabolism, and protein synthesis [61–63]. mTOR is present in two complexes, mTORC1 and mTORC2 [18, 62]. Rictor is a key regulatory/structural subunit of mTORC2 [63]. Recent studies indicate that Rictor (mTORC2) signaling regulates a variety of cellular processes, such as cell metabolism and survival, under conditions of oxidative stress [18, 61, 63, 64]. Herein, we reported that seminal Rictor expression was higher, but the ratio of phospho-Rictor/Rictor was lower in AST than in NOR. Based on the above evidence, we hypothesized that oxidative stress affects the seminal microenvironment by changing biochemical compositions and energy metabolism through the AMPK/mTORC2 signaling pathway, leading to impaired sperm activity and stability, and ultimately causes asthenozoospermia. But the specific mechanism requires further validation.

## 5. Conclusion

The ATR-FTIR spectrum reported in this study reveals that the reduced sperm motility of idiopathic asthenozoospermia is related to changes in the structure, property, and content of seminal plasma biomolecules. In addition, changes in the level of peptide aggregation, protein phosphorylation, unsaturated fatty acid, and lipid saturation affect sperm motility by regulating seminal plasma metabolism in AST. Oxidative stress can directly or indirectly affect seminal plasma metabolism by activating the AMPK/mTOR2 signaling pathway, thus leading to lower sperm motility. The limitation of the study is that there lacks a functional study, so it needs further researches to validate the specific correlations. Our findings provide a theoretical and experimental basis for subsequent research on the pathogenesis of asthenozoospermia. But it still needs further validation by other quantitative analysis, such as mass spectrometry and nuclear magnetic resonance.

## Data Availability

The data used to support the findings of this study are available from the corresponding author upon request.

## Supplementary Materials

Supplementary Materials Supplemental Table 1: baseline demographic and clinical characteristics of the subjects. Supplemental Figure 1: principal component analysis of ATR-FTIR spectral data. The results of PCA show the contribution percentage of five principal components applied to different seminal plasma types: NOR, OLI, and AST. Supplemental Figure 2: variations for the ATR-FTIR absorption bands of NOR *vs.* AST and OLI *vs.* AST. Supplemental Figure 3: the expression levels of nonsignificant target proteins in the AMPK/mTOR signaling pathway between NOR and AST.
