# Supplementary material for: The Activated AMPK/mTORC2 Signaling Pathway Associated with Oxidative Stress in Seminal Plasma Contributes to Idiopathic Asthenozoospermia
Source: Oxid Med Cell Longev. 2022 Jun 8;2022:4240490. doi: 10.1155/2022/4240490 (PMC9200551; doi:10.1155/2022/4240490)
Supplement: Supplementary Materials — Supplemental Table 1: baseline demographic and clinical characteristics of the subjects. Supplemental Figure 1: principal component analysis of ATR-FTIR spectral data. The results of PCA show the contribution percentage of five principal components applied to different seminal plasma types: NOR, OLI, and AST. Supplemental Figure 2: variations for the ATR-FTIR absorption bands of NOR vs. AST and OLI vs. AST. Supplemental Figure 3: the expression levels of nonsignificant target proteins in the AMPK/mTOR signaling pathway between NOR and AST. [file 4240490.f1.zip › Supplemental Figure 1-3.docx]

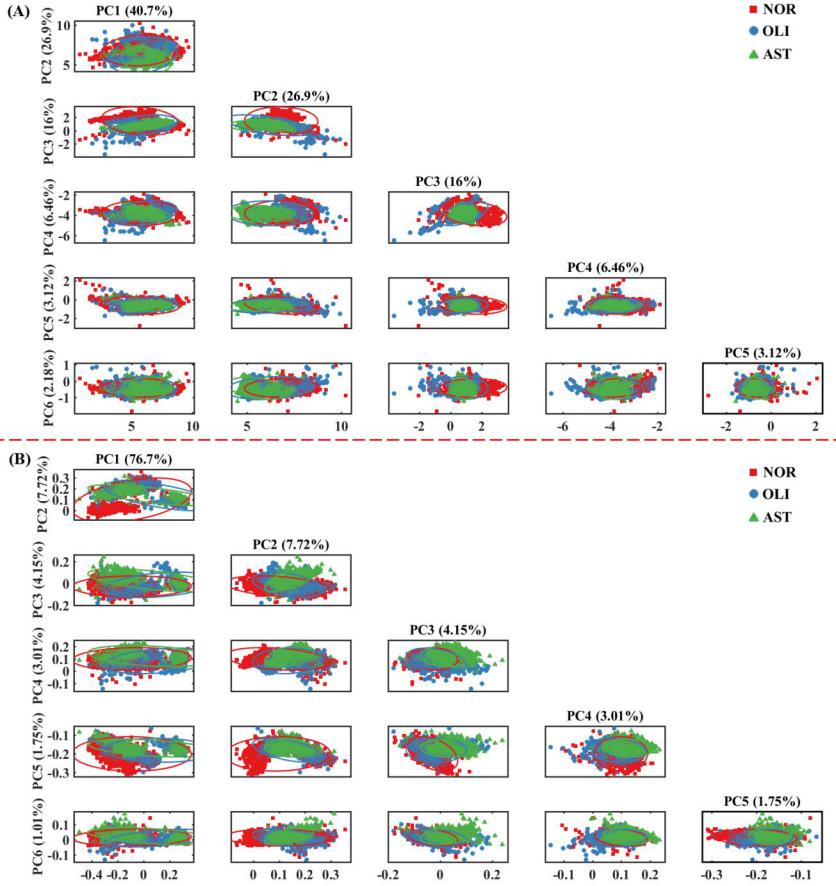


**SUPPLEMENTAL FIGURE 1:** Principal component analysis of ATR-FTIR spectral data. The results of PCA showing the contribution percentage of five principal components applied to different seminal plasma types: NOR (red square), OLI (blue square), and AST (green square). The resultant spectra included two regions of interest at 1800–900 cm^−1^ (**A**) and 3100–2800cm^−1^ (**B**). NOR: normozoospermia; OLI: idiopathic oligozoospermia; AST: idiopathic asthenozoospermia. PCA: principal component analysis.


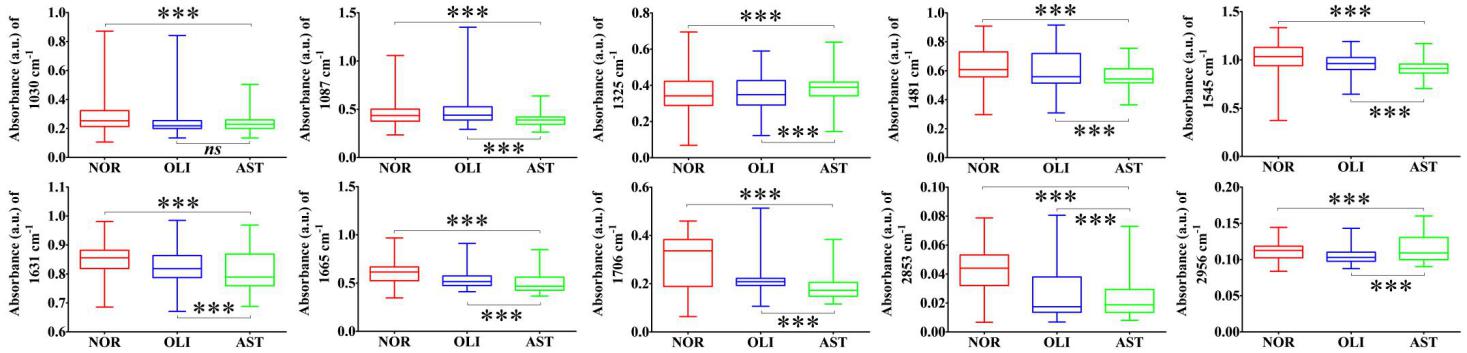


**SUPPLEMENTAL FIGURE 2:** Variations for the ATR-FTIR absorption bands of NOR *vs.* AST and OLI *vs.* AST. NOR: normozoospermia; OLI: idiopathic oligozoospermia; AST: idiopathic asthenozoospermia. Statistical descriptive analysis was performed through box plot diagram, ****P*<0.001 *vs*. corresponding seminal plasma type by one-way ANOVA with Fisher's LSD or Dunnett’s T3 post-hoc test. 1030 cm^−1^ (glucose), 1087 cm^−1^ (symmetric PO_2_^−^ stretching of DNA and RNA), 1325 cm^−1^ (Amide ΙΙΙ), 1481 cm^−1^ (protein conformation), 1545 cm^−1^ (Amide ΙΙ), 1631 cm^−1^ (Amide Ι: β-sheet), 1665 cm^−1^ (Amide Ι), 1706 cm^−1^ (C=O stretching of lipids), 2853 cm^−1^ (*νs* CH_2_ of lipids), 2956 cm^−1^ (asymmetric stretching of CH_3_).


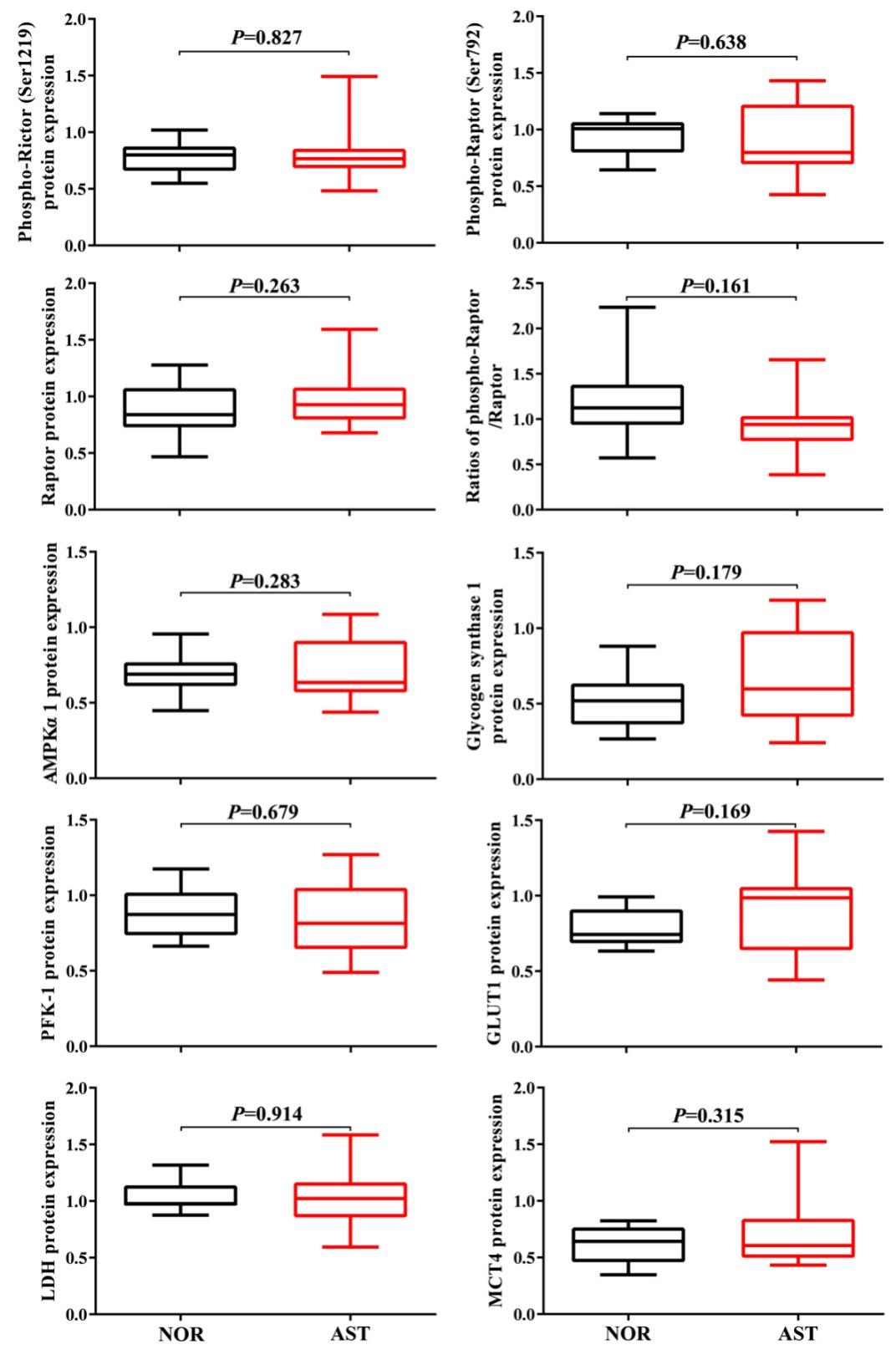


**SUPPLEMENTAL FIGURE 3**: Box plot diagram showing the expression levels of phospho-Rictor (Ser1219), phospho-Raptor (Ser792), Raptor, phospho-Raptor/Raptor, AMPKα1, Glycogen synthase 1, PFK-1, GLUT1, LDH, and MCT4. GLUT1: glucose transporter 1; LDH: lactate dehydrogenase; PFK-1:phosphofructoki-nase-1; MCT4: monocarboxylate transporter 4; NOR: normozoospermia; AST: idiopathic asthenozoospermia.
