# Supplementary material for: The Activated AMPK/mTORC2 Signaling Pathway Associated with Oxidative Stress in Seminal Plasma Contributes to Idiopathic Asthenozoospermia
Source: Oxid Med Cell Longev. 2022 Jun 8;2022:4240490. doi: 10.1155/2022/4240490 (PMC9200551; doi:10.1155/2022/4240490)
Supplement: Supplementary Materials — Supplemental Table 1: baseline demographic and clinical characteristics of the subjects. Supplemental Figure 1: principal component analysis of ATR-FTIR spectral data. The results of PCA show the contribution percentage of five principal components applied to different seminal plasma types: NOR, OLI, and AST. Supplemental Figure 2: variations for the ATR-FTIR absorption bands of NOR vs. AST and OLI vs. AST. Supplemental Figure 3: the expression levels of nonsignificant target proteins in the AMPK/mTOR signaling pathway between NOR and AST. [file 4240490.f1.zip › Supplemental Table 1.docx]

**SUPPLEMENTAL TABLE 1:** Baseline demographic and clinical characteristics of the subjects.

| **Parameters** | **NOR**  **(n=70)** | **OLI**  **(n=57)** | **AST**  **(n=53)** | ***P*^a^** | ***P*^b^** | ***P*^c^** |
| --- | --- | --- | --- | --- | --- | --- |
| **Sociodemographic parameters** |  |  |  |  |  |  |
| Age (year, median, IQR) | 30.00 (24.00, 32.00) | 30.00 (27.00, 33.25) | 29.00 (26.00, 29.00) | 0.949 | 0.993 | 0.917 |
| BMI (kg/m^2^, median, IQR) | 24.37 (19.26, 28.85) | 25.35 (18.17, 29.34) | 23.65 (18.73, 30.15) | 0.211 | 0.324 | 0.247 |
| Education |  |  |  |  |  |  |
| Less than college (%) | 25.71 | 40.35 | 30.08 | 0.667^d^ | 0.783^d^ | 0.921^d^ |
| College (%) | 42.86 | 36.84 | 37.74 |  |  |  |
| Graduate school (%) | 21.43 | 22.81 | 30.19 |  |  |  |
| Smoking habit |  |  |  |  |  |  |
| Never (%) | 11.43 | 10.53 | 7.55 | 0.095^d^ | 0.220^d^ | 0.745^d^ |
| Former (%) | 22.86 | 36.84 | 45.28 |  |  |  |
| Current (%) | 65.71 | 52.63 | 60.38 |  |  |  |
| Drinking habit |  |  |  |  |  |  |
| Never (%) | 1.43 | 0 | 0 | 0.350^d^ | 0.623 | 0.177^d^ |
| Former (%) | 48.57 | 45.61 | 58.49 |  |  |  |
| Current (%) | 50.00 | 54.39 | 41.51 |  |  |  |
| Sedentary time (hours/day, median, IQR) | 6.60(4.80, 7.79) | 6.11(4.83, 7.60) | 5.31(4.99, 7.77) | 0.325 | 0.060 | 0.380 |
| Sleep duration (hours/day, median, IQR) | 7.51(7.03, 8.51) | 7.14(6.82, 7.92) | 7.55(7.05, 8.03) | 0.072 | 0.279 | 0.514 |
| **Clinical and laboratory parameters** |  |  |  |  |  |  |
| Ejaculate volume (ml, median, IQR) | 3.40 (3.00, 4.20) | 3.00 (2.58, 3.53) | 3.30 (2.90, 4.40) | 1.000 | 0.005 | 0.127 |
| Sperm concentration (millions/ml, median, IQR) | 43.70 (33.30, 68.30) | 7.07 (3.18, 9.55) | 25.70 (19.85, 32.09) | <0.001 | <0.001 | <0.001 |
| Total sperm count (millions, median, IQR) | 161.70 (114.84, 244.50) | 22.10 (8.74, 28.08) | 95.60 (60.85, 120.82) | <0.001 | <0.001 | <0.001 |
| Total motility percentage (median, IQR) | 67.50 (57.16, 75.46) | 57.20 (48.50, 61.84) | 30.20 (25.95, 33.80) | <0.001 | <0.001 | <0.001 |
| Progressive motility percentage (median, IQR) | 56.40 (48.40, 65.80) | 45.42 (39.24, 50.33) | 20.80 (17.00, 25.87) | <0.001 | <0.001 | <0.001 |
| Non-Progressively motility percentage (median, IQR) | 9.40 (6.92, 11.85) | 10.40 (6.73, 14.83) | 7.50 (5.10, 10.85) | 0.036 | 0.439 | 0.022 |
| VCL (μm/sec, median, IQR) | 58.53 (45.86, 75.95) | 55.45 (37.17, 79.60) | 54.30 (38.52, 70.05) | 0.395 | 0.993 | 0.545 |
| MAD (degree, median, IQR) | 59.00 (48.75, 101.79) | 71.55 (50.87, 101.44) | 88.94 (56.38, 129.33) | 0.002 | 0.194 | 0.144 |
| LIN (%, median, IQR) | 59.70 (51.85, 67.06) | 52.75 (46.30, 60.01) | 53.40 (43.05, 62.98) | <0.001 | 0.001 | 0.986 |
| VSL (μm/sec, median, IQR) | 35.52 (29.74, 43.45) | 30.12 (23.90, 42.75) | 27.70 (23.70, 40.65) | 0.001 | 0.030 | 0.351 |
| ALH (μm, median, IQR) | 3.52 (2.52, 5.10) | 3.55 (2.59, 5.25) | 3.50 (2.83, 4.11) | 0.790 | 0.221 | 0.234 |
| WOB (%, median, IQR) | 67.48 (62.25, 73.39) | 62.80 (55.15, 69.44) | 61.30 (54.50, 69.87) | <0.001 | 0.004 | 0.568 |
| VAP (μm/sec, median, IQR) | 40.20 (32.53, 50.60) | 34.94 (26.86, 49.50) | 32.10 (27.04, 45.20) | 0.006 | 0.100 | 0.408 |
| BCF (Hz, median, IQR) | 4.89 (4.50, 5.34) | 4.57 (3.90, 5.53) | 4.40 (4.10, 5.23) | 0.030 | 0.038 | 0.974 |
| STR (%, median, IQR) | 86.54 (82.00, 89.76) | 79.70 (75.26, 84.97) | 81.00 (76.85, 87.87) | 0.001 | <0.001 | 0.212 |
| Normal morphology (%, median, IQR) | 5.04(4.59, 5.71) | 2.90(2.58, 3.06) | 3.67(3.53, 4.01) | <0.001 | <0.001 | <0.001 |
| DNA fragmentation index (%, median, IQR) | 20.89(19.97, 22.50) | 38.09(35.01,40.65) | 42.11(37.35, 44.96) | <0.001 | <0.001 | 0.002 |
| Acrosome-reacted sperm (%, median, IQR) | 24.90(16.50, 36.85) | 20.30(5.20, 24.70) | 16.40 (8.30, 25.58) | <0.001 | 0.012 | 1.000 |
| NAG-Total (mU/ejaculate, median, IQR) | 62.51(38.10, 88.77) | 49.68 (32.47,78.50) | 56.81(48.13, 66.44) | 0.835 | 0.124 | 0.102 |
| Elastase (ng/ml, median, IQR) | 620.61(309.42, 947.435) | 644.16(304.75, 1352.01) | 663.21(319.49, 1274.07) | 0.486 | 0.160 | 0.515 |
| Zn-Total (µmol/ejaculate, median, IQR) | 8.88 (5.51, 12.19) | 7.02(5.79, 11.81) | 7.49(5.10, 11.54) | 0.441 | 0.712 | 0.697 |
| Fructose-Total (µmol/ejaculate, median, IQR) | 46.00(30.46, 72.82) | 47.13(38.21, 67.17) | 40.21(25.48, 66.26) | 0.126 | 0.667 | 0.063 |
| Acrosin (µIU/10^6^ sperm, median, IQR) | 104.82(71.26, 143.65) | 92.82(68.55, 135.35) | 73.70(40.79, 112.14) | <0.001 | 0.675 | 0.003 |
| Serum FSH (IU/L, median, IQR) | 5.22(4.78, 5.60) | 5.28(4.66, 5.71) | 5.12(4.73, 5.63) | 0.281 | 0.315 | 0.929 |
| Serum LH (IU/L, median, IQR) | 4.30(3.92, 4.58) | 4.17(3.93, 5.70) | 4.23(3.94, 4.61) | 0.770 | 0.260 | 0.439 |
| Serum T (nmol/L, median, IQR) | 12.26(11.27, 13.26) | 12.60(11.48, 13.72) | 12.40(11.05, 13.49) | 0.806 | 0.253 | 0.404 |

NOR: normozoospermia; OLI: idiopathic oligozoospermia; AST: idiopathic asthenozoospermia; VCL: curvilinear velocity; MAD: mean angle of deviation; LIN: linearity; VSL: straight-line velocity; ALH: amplitude of lateral head displacement; WOB: wobble; VAP: average path velocity; BCF: beat cross frequency; STR: straightness; NAG: neutral alpha-glucosidase; FSH: follicle stimulating hormone; LH: luteinizing hormone; T: testosterone. The differences among the groups were analyzed by one-way ANOVA with the Fisher's LSD or Dunnett’s T3 post-hoc test. ^a^ AST *vs.* NOR; ^b^ OLI *vs.* NOR; ^c^ AST *vs.* OLI. ^d^ Two-sided χ2 test for the frequency distribution of selected variables among the groups. *P* < 0.05 indicates statistically significant.
